# Evaluation of left ventricular and left atrial volumetric function from native MR multislice 4D flow magnitude data

**DOI:** 10.1007/s00330-023-10017-3

**Published:** 2023-08-15

**Authors:** Clemens Reiter, Gert Reiter, Corina Kräuter, Daniel Scherr, Albrecht Schmidt, Michael Fuchsjäger, Ursula Reiter

**Affiliations:** 1 Division of General Radiology, Department of Radiology, Medical University of Graz, Auenbruggerplatz 9/P, 8036 Graz, Austria; 2 Division of Neuroradiology, Vascular and Interventional Radiology, Department of Radiology, Medical University of Graz, Graz, Austria; 3 Division of Cardiology, Department of Internal Medicine, Medical University of Graz, Graz, Austria; 4 Research and Development, Siemens Healthcare Diagnostics GmbH, Graz, Austria

**Keywords:** Magnetic resonance imaging, Cardiovascular system, Diagnostic imaging, Heart function tests, Validation study

## Abstract

**Objectives:**

To assess the feasibility, precision, and accuracy of left ventricular (LV) and left atrial (LA) volumetric function evaluation from native magnetic resonance (MR) multislice 4D flow magnitude images.

**Materials & Methods:**

In this prospective study, 60 subjects without signs or symptoms of heart failure underwent 3T native cardiac MR multislice 4D flow and bSSFP-cine realtime imaging. LV and LA volumetric function parameters were evaluated from 4D flow magnitude (4D flow-cine) and bSSFP-cine data using standard software to obtain end-diastolic volume (EDV), end-systolic volume (ESV), ejection-fraction (EF), stroke-volume (SV), LV muscle mass (LVM), LA maximum volume, LA minimum volume, and LA total ejection fraction (LATEF). Stroke volumes derived from both imaging methods were further compared to 4D pulmonary artery flow-derived net forward volumes (NFV). Methods were compared by correlation and Bland-Altman analysis.

**Results:**

Volumetric function parameters from 4D flow-cine and bSSFP-cine showed high to very high correlations (r = 0.83-0.98). SV, LA volumes and LATEF did not differ between methods. LV end-diastolic and end-systolic volumes were slightly underestimated (EDV: –2.9 ± 5.8 mL; ESV: -2.3 ± 3.8 mL), EF was slightly overestimated (EF: 0.9 ± 2.6%), and LV mass was considerably overestimated (LVM: 39.0 ± 11.4 g) by 4D flow-cine imaging. SVs from both methods correlated very highly with NFV (r = 0.91 in both cases) and did not differ from NFV.

**Conclusion:**

Native multislice 4D flow magnitude data allows precise evaluation of LV and LA volumetric parameters; however, apart from SV, LV volumetric parameters demonstrate bias and need to be referred to their respective normal values.

**Clinical relevance statement:**

Volumetric function assessment from native multislice 4D flow magnitude images can be performed with routinely used clinical software, facilitating the application of 4D flow as a one-stop-shop functional cardiac MR exam, providing consistent, simultaneously acquired, volume and flow data.

**Key points:**

• *Native multislice 4D flow imaging allows evaluation of volumetric left ventricular and atrial function parameters.*

• *Left ventricular and left atrial function parameters derived from native multislice 4D flow data correlate highly with corresponding standard cine-derived parameters.*

• *Multislice 4D flow-derived volumetric stroke volume and net forward volume do not differ.*

## Introduction

Cardiac magnetic resonance (MR) two-dimensional (2D) balanced steady-state free precession (bSSFP) cine imaging has been established as the standard reference technique for the assessment of ventricular systolic function and myocardial mass [1–3]. Time-resolved, three-dimensional (3D), three-directional MR phase contrast (4D flow) imaging provides – in addition to 3D velocity fields – three-dimensional anatomical (magnitude) cine images. While numerous novel velocity-related cardiac functional 4D flow parameters have been explored [4–6], the volumetric assessment of cardiac function using 4D flow magnitude images has rarely been investigated [7–11]. However, acquisition of both volumetric function and flow-based hemodynamic parameters from one measurement would not only allow to simplify and shorten MR imaging protocols but would also enable the comparison of parameters without physiological cycle-to-cycle variations. The latter aspect could significantly improve cross-check evaluation between volumetric and flow parameters, e.g. when applying the conservation-of-mass principle to evaluate mitral valve regurgitation volumes using LV volumetric stroke volume and aortic net flow volume, or when controlling segmentations quality [6, 12, 13].

Compared to bSSFP-cine sequences, 4D flow magnitude images are based on fast low-angle shot (FLASH) readout, which intrinsically has lower blood-to-myocardium contrast [14, 15]. Furthermore, 4D flow techniques are typically implemented as 3D sequences, which suffer from reduced in-flow blood enhancement and consequently lower blood-to-myocardium contrast than 2D-based measurements [6, 14, 16]. To address the latter problem in 3D acquisitions, data for volumetric evaluation from 4D flow measurements have been acquired after application of gadobenate dimeglumine, gadopentetate dimeglumine, or gadoterate meglumine [9–11], or following administration of ferumoxytol (used off-label) as a contrast agent [7, 8]. This has allowed the extraction of left and right ventricular volumetric function parameters comparable to standard 2D bSSFP-cine-derived measurements [7–11]. However, because of the controversy regarding the safety of gadolinium and ferumoxytol as contrast agents, their use, when not indicated by the referral diagnosis, is difficult to justify [17, 18].

Using a multislice time-resolved 2D phase contrast sequence with three-directional velocity encoding to acquire multislice 4D flow data, the corresponding native (non-contrast enhanced) magnitude series could potentially be applied for volumetric function evaluation. Therefore, the aim of the present study was to investigate the applicability of native multislice 4D flow magnitude images for the evaluation of left ventricular (LV) and left atrial (LA) volumetric function parameters, and to validate the results by comparison with bSSFP-cine imaging-derived LV and LA volumetric function parameters as well as 4D flow-derived net forward volume.

## Materials and methods

### Study population

**Fig. 1:**
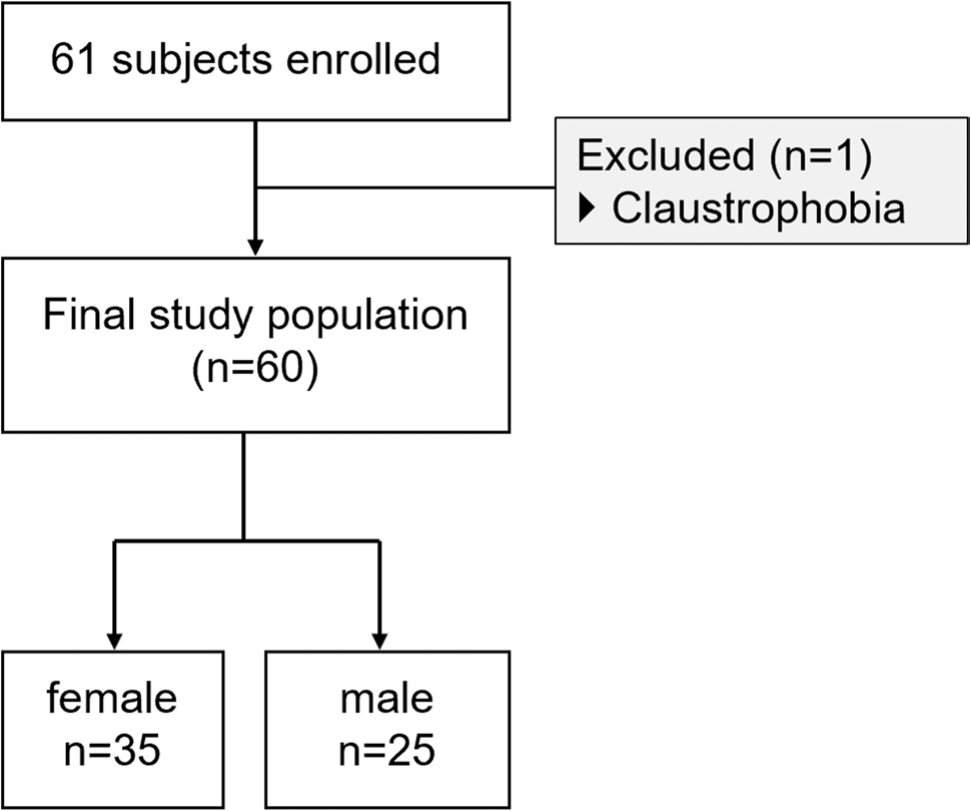
Study flowchart

This prospective study was approved by the local ethical review board and complied with the Declaration of Helsinki. All participants provided written informed consent. Sixty-one subjects without signs or symptoms of heart failure were recruited between October 2016 and March 2017 for native cardiac MR imaging. Exclusion criteria were abnormal heart rhythm, known cardiac shunts, and contraindications to MR. One subject did not undergo cardiac MR imaging because of claustrophobia. Therefore, 60 subjects were included in the data analysis. Figure 1 presents the subject flowchart for the study.

### Cardiac magnetic resonance imaging

All subjects underwent comprehensive electrographically (ECG)-gated 3T cardiac MR imaging (Magnetom Skyra, Siemens Healthineers) in the supine position using a phased-array 18-channel body matrix and a spine matrix coil. The protocol included free breathing bSSFP-cine realtime and 4D flow imaging.

bSSFP-cine realtime series were acquired in LV 2-chamber, 4-chamber, and 3-chamber views, as well as in a stack of contiguous slices in short-axis orientation covering the LV cavity. To cover all phases of the cardiac cycle, series were acquired for approximately 1.5 heartbeats. Typical protocol parameters were as follows: spatial resolution, 2.3 × 3.9 × 7.0 mm^3^ for long-axis and 2.5 × 4.2 × 8.0 mm^3^ for short-axis images; echo/repetition time, 1.1/2.5 ms; flip angle, 40°; parallel acquisition factor, 3; temporal resolution, 36 ms. Cardiac shimming and transmission frequency optimization were employed to minimize bSSFP-related dark band artifacts.

Multislice 4D flow data were acquired in 3-chamber orientation using a retrospectively ECG-gated, FLASH-based 2D phase-contrast sequence with simple three-directional velocity encoding [19, 20] covering the heart. Typical protocol parameters were as follows: spatial resolution, 1.8 x 2.5 x 4 mm^3^; echo/repetition time, 3.1/5.2 ms; parallel acquisition factor, 2; temporal resolution = 41.8 ms interpolated to 30 cardiac phases; number of averages, 2; 21-39 gapless slices; 36-62 heartbeats per slice; typical scan time, 22 minutes. Velocity encoding between 100 and 140 cm/s was chosen to prevent aliasing in the cardiac chambers. Arrythmia rejection was used to suppress artefacts from ECG miss-triggering or irregular heartbeats.

### Image analysis

#### Preprocessing of 4D flow magnitude data and image quality scoring

**Fig. 2:**
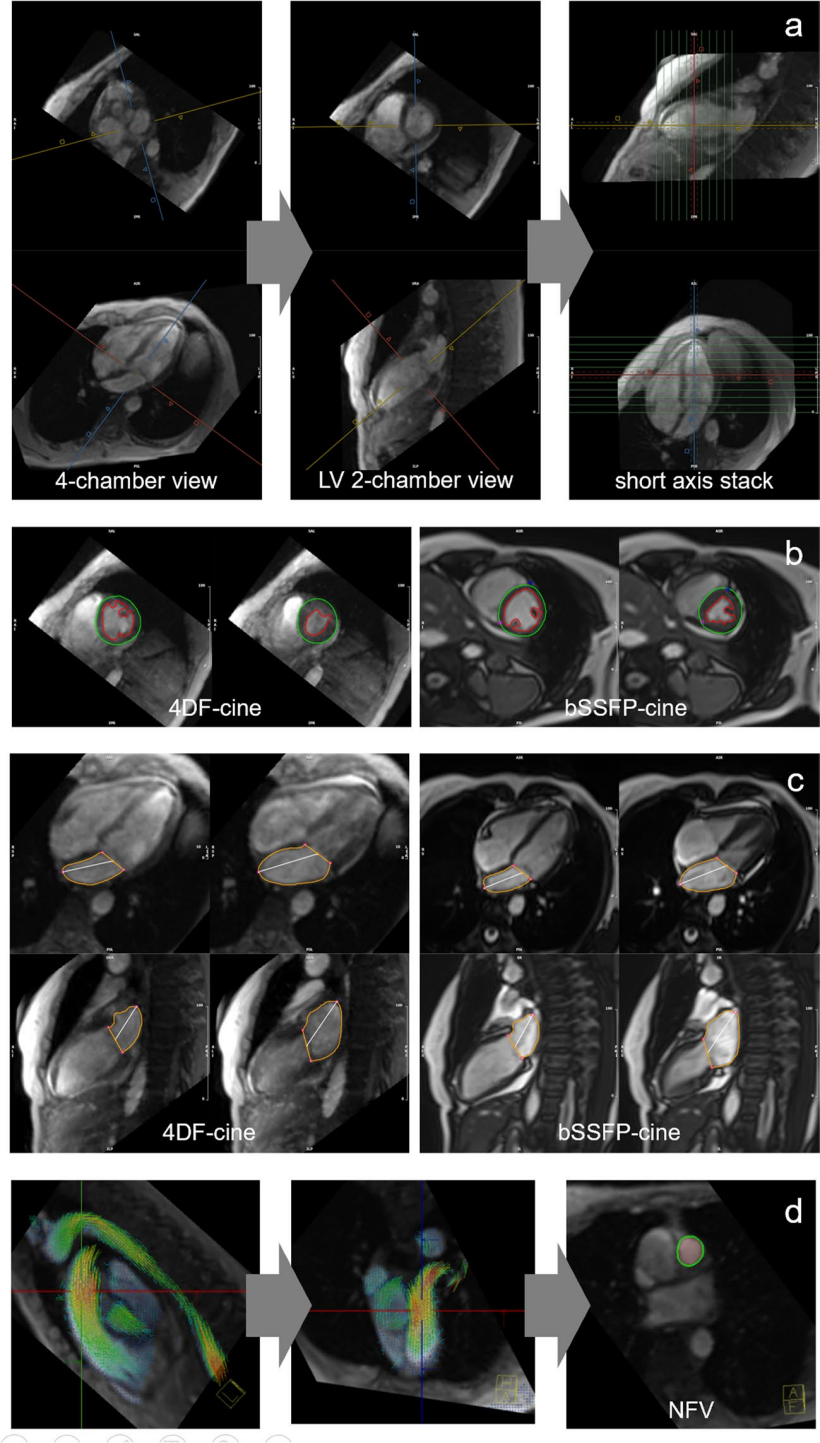
Pre-processing and evaluation of native 4D flow magnitude data. Multiplanar reconstruction of 4D flow-cine series in 4-chamber, 2-chamber and short-axis views (**a**): The 4-chamber view was reconstructed through the center of the mitral and tricuspid valve in a basal short-axis cut plane (left panel). The LV 2-chamber view was orientated parallel to the left-right ventricular insertion points positioned in the center of the LV cavity (mid panel). Stacks of short-axis images covering the LV were reconstructed in the end-diastolic phase (right panel). LV segmentation in short-axis images with exclusion of the papillary muscles and trabeculae from the blood pool from 4D flow-cine (**b**, left panel) and bSSFP-cine (**b**, right panel). LA segmentation in long-axis images from 4D flow-cine (**c**, left panel) and bSSFP-cine (**c**, right panel). Multiplanar reconstruction and segmentation of pulmonary artery cross section for evaluation of pulmonary artery net forward volume (NFV) (**d**)

4D flow magnitude datasets were multiplanar reconstructed in LV 2-chamber and 4-chamber view cut planes, as well as a stack of 11-14 contiguous short-axis slices (slice thickness, 8 mm) covering the entire LV cavity using standard software (cvi42, Circle Cardiovascular imaging) to obtain 4D flow-cine series for further evaluation (Fig. 2a).

Image quality of end-systolic and end-diastolic 4D flow- and bSSFP-cine images in the stack of cine short-axis as well as in cine long-axis images were evaluated by an experienced reader (C.R., 7 years of experience) using the following 5-point Likert scale based on the visibility of endocardial borders [21, 22]: 5 = excellent (all borders can clearly be delineated), 4 = good (mild artefacts, borders have to be interpolated minimally), 3 = adequate (moderate artefacts, borders have to interpolated over short distances), 2 = fair (significant artefacts, borders have to be interpolated for larger parts of contours), 1 = inadequate (borders cannot be reliably identified).

#### Volumetric evaluation

LV and LA volumetric function parameters were evaluated from 4D flow-cine and bSSFP-cine series using standard software (cvi42, Circle Cardiovascular imaging). Heart rate was determined as an average throughout the measurements. Whereas on 4D flow-cine images contours were drawn manually, for bSSFP-cine images the segmentations of LV and LA automatically suggested by the software were inspected and corrected where necessary.

LV end-diastole (ED) and end-systole (ES) were each defined visually in a midventricular short-axis slice as the phases with the largest and smallest LV cross-sectional areas, respectively [23, 24]. LV end-diastolic volume (EDV), end-systolic volume (ESV), stroke volume (SV), ejection fraction (EF) and LV myocardial mass (LVM) were evaluated by segmentation of the ED and ES endo- and epicardial short-axis contours. Window/level was adjusted to optimize the blood-to-myocardial contrast. The most basal LV short-axis slice was selected as the one with at least 50% of the LV cavity surrounded by myocardium [3]. Papillary muscles and trabeculations were excluded from the LV blood pool, while the LV outflow tract was included in the LV cavity (Fig. 2b). A discrepancy > 5% between end-diastolic and end-systolic LVM was used to identify incorrect segmentation, which was then addressed by correction of the endo- and/or epicardial borders as needed. The reported LVM is the average of the systolic and diastolic results.

LA minimal volume (LAV_min_), LA maximal volume (LAV_max_), and the total LA ejection fraction (LATEF) were evaluated using the bi-planar area-length method [25]. Volumes were derived from manual segmentation of the LA cavity in the cardiac phases with the largest and smallest LA cross-sectional areas with a closed mitral valve as visualized in the LV 2- and 4-chamber view series. The mitral valve plane was approximated by a straight line. The LA appendage was included, and pulmonary veins were excluded from the LA cavity (Fig. 2c) [26].

To investigate the intra- and inter-observer variability of volumetric function parameters from 4D flow-cine and bSSFP-cine measurements, the first 10 consecutive female and 10 consecutive male subjects were evaluated twice by one observer and once by a second observer (C.R. and U.R., 7 and 20 years of experience, respectively). Data were analyzed blinded to prior evaluations.

#### 4D flow phase contrast evaluation

4D flow velocity fields were analyzed employing prototype software (4Dflow, Siemens Healthineers). LV and LA velocity vector fields were visually inspected to detect unknown cardiac shunts or significant valve regurgitations. Pulmonary artery net forward volume (NFV) was assessed from the phase-offset-corrected 4D flow velocity field by multiplanar reconstruction of an evaluation plane through the main pulmonary artery above the pulmonary valve. Pulmonary artery cross-sectional area was automatically segmented and manually corrected if necessary (Fig. 2d).

### Statistical analysis

Statistical analysis was performed using SPSS® (SPSS Software v28). Distributions of parameters are specified as means and standard deviations (SD) as well as modus in case of image scores; 95% confidence intervals are given in brackets.

Normality of distributions was tested with the Shapiro–Wilk test. As appropriate, differences of non-paired samples were compared by t-test or Mann–Whitney U test, differences of paired samples by paired t-test or Wilcoxon signed-rank test. Pearson correlation (r) and Bland-Altman analysis were employed to study the relationship between continuous parameters from 4D flow and bSSFP-cine measurements. Two-way mixed effect model, single measure, absolute agreement intra-class correlation coefficients (ICC) were used to quantify inter- and intra-observer variability. Correlations were classified according to the correlation coefficients as low (0.3–0.5), moderate (0.5–0.7), high (0.7–0.9), or very high (0.9–1.0) [27].

A *p *value < 0.05 was regarded as statistically significant. For ICCs, non-overlapping confidence intervals were regarded as indicating a significant difference.

## Results

The baseline characteristics of the study subjects are summarized in Table 1. None of the subjects analyzed demonstrated a cardiac shunt or significant valve regurgitation in the 4D flow velocity field.

**Table 1:** Baseline characteristics of the study population. BSA, body surface area; sBP, systolic blood pressure; dBP, diastolic blood pressure

parameter	total	female	male	*p*
*demographic data*				
subjects (number)	60	35	25	
age (years)	61 ± 9	60 ± 8	62 ± 10	0.371
size (cm)	171 ± 9	165 ± 7	178 ± 7	< 0.001
weight (kg)	75 ± 14	67 ± 10	85 ± 12	< 0.001
BSA (m^2^)	1.87 ± 0.21	1.75 ± 0.15	2.05 ± 0.17	< 0.001
sBP (mmHg)	135 ± 17	130 ± 17	143 ± 15	0.003
dBP (mmHg)	76 ± 9	73 ± 10	80 ± 6	0.001
*medical history*				
no prior medical history	23 (38%)	9 (26%)	14 (56%)	
hypertension	16 (27%)	9 (26%)	7 (28%)	
hypothyroidism	19 (32%)	17 (49%)	2 (8%)	
hyperlipidemia	5 (8%)	4 (11%)	1 (4%)	
osteoporosis	6 (10%)	6 (17%)		
gout	3 (5%)	1 (3%)	2 (8%)	
rheumatoid arthritis	2 (3%)	2 (6%)		

### Image quality

**Fig. 3:**
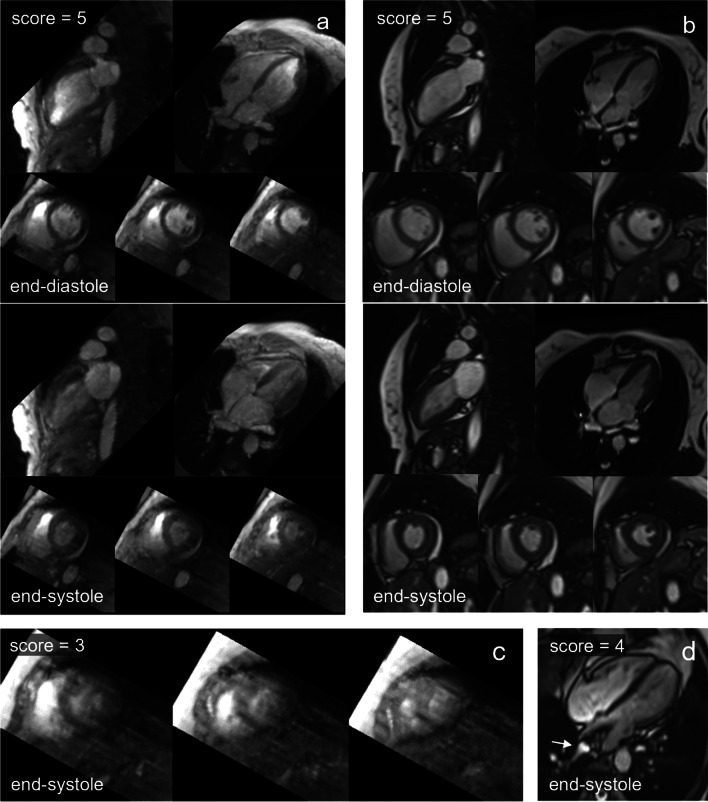
Representative examples of images with adequate to excellent image quality scores. End-diastolic and end-systolic LV 2-chamber, 4-chamber and short-axis images in 4D flow-cine (**a**) and bSSFP-cine (**b**) series with excellent quality scores. 4D flow-cine short-axis images of adequate quality (**c**). bSSFP-cine images (3T off-resonance band in the left atrium is marked by arrow) of good quality (**d**).

All evaluated images received a quality score ≥ 3. Examples with overall excellent image quality scores are shown in Fig. 3a and b. Average image quality scores for 4D flow-cine and bSSFP-cine images are given in Table 2 and demonstrate differences in systolic short-axis as well as systolic 4-chamber view images. Representative examples of systolic 4D flow-cine short-axis and systolic 4-chamber bSSFP-cine images with lower image quality scores are shown in Figures 3c and 3d, respectively.

**Table 2:** Image quality scores for MSL-4D flow-cine and bSSFP-cine images. Data are reported as mean and standard deviations (SD) together with the modal value given in parentheses. The *p* value corresponds to the Wilcoxon rank-sum test between 4D flow-cine and bSSFP-cine quality scores. *p*, significance level; 4ch, 4-chamber view; 2ch, left ventricular 2-chamber view; SA, short-axis view

parameter	4D flow-cine	bSSFP-cine	*p*
4ch diastolic	4.98 ± 0.13 (5.0)	4.92 ± 0.28 (5.0)	0.104
4ch systole	4.98 ± 0.13 (5.0)	4.92 ± 0.28 (5.0)	0.046
2ch diastolic	4.98 ± 0.13 (5.0)	5.00 ± 0.00 (5.0)	0.325
2ch systolic	4.97 ± 0.18 (5.0)	4.98 ± 0.13 (5.0)	0.325
SA diastolic	4.95 ± 0.29 (5.0)	5.00 ± 0.00 (5.0)	0.161
SA systolic	3.88 ± 0.52 (4.0)	5.00 ± 0.00 (5.0)	< 0.001

### Volumetric function parameters

**Fig. 4:**
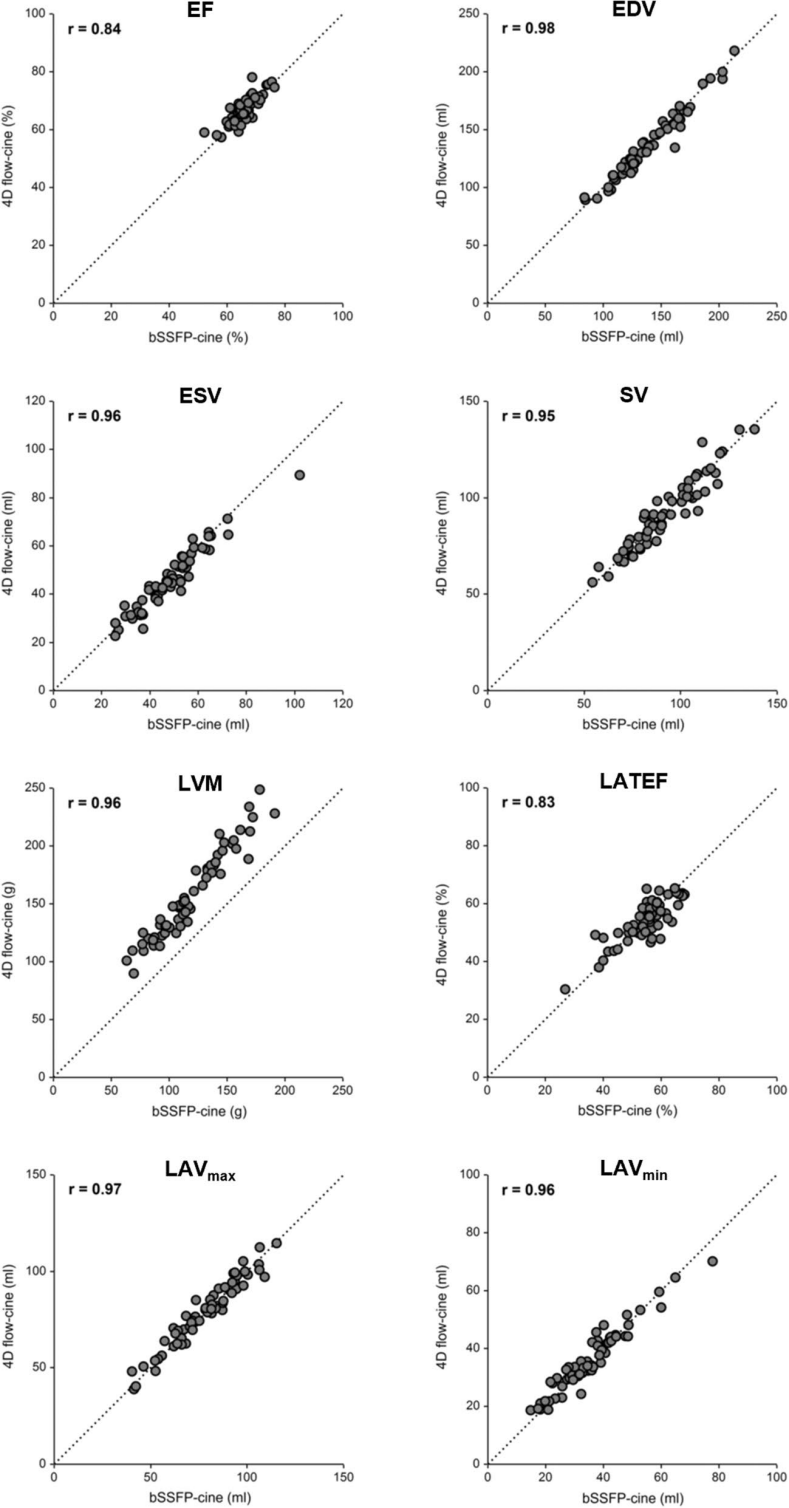
Scatter-plots of LV and LA volumetric function parameters from 4D flow-cine and bSSFP-cine images. Correlation coefficients (r) and lines of identity (dotted line) are given

**Fig. 5:**
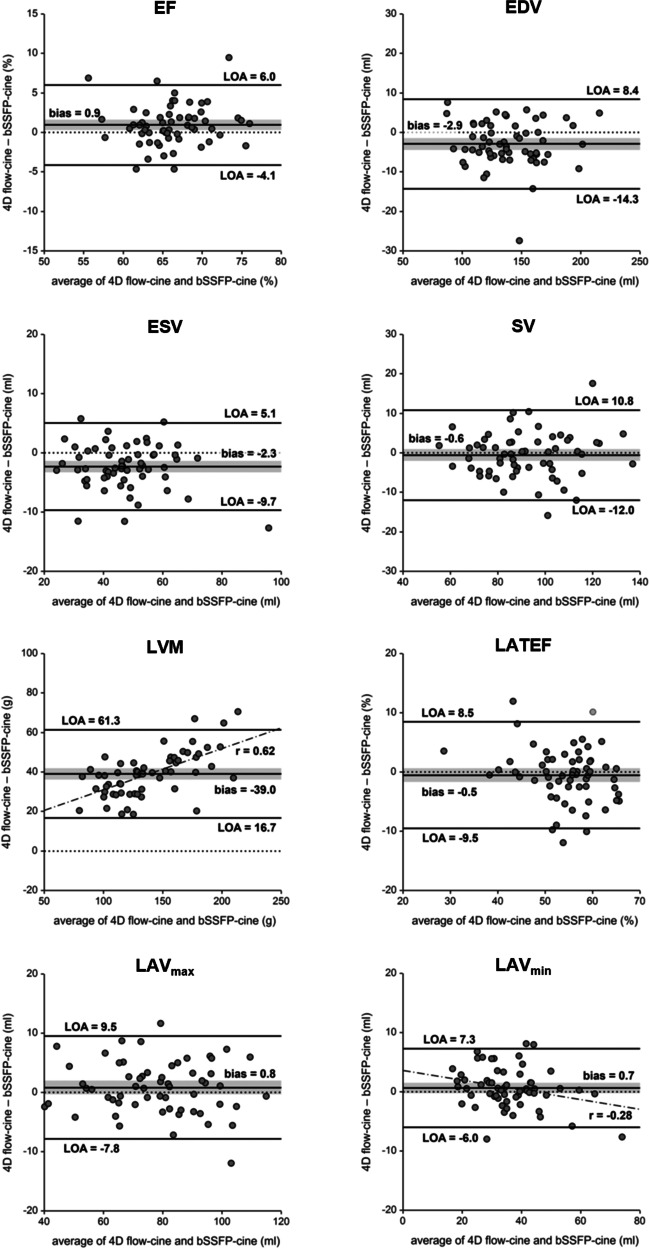
Bland-Altman plots comparing LV and LA volumetric function parameters from 4D flow-cine and bSSFP-cine images. The grey bar indicates the 95% confidence intervals of bias. Significant correlations between differences and averages of a parameter are indicated by the drawn regression line together with the correlation coefficient (r). LOA, limits of agreement

The average heart rate during 4D flow (68 ± 10 min^-1^) and bSSFP-cine imaging (67 ± 10 min^-1^) did not differ (*p* = 0.357). There were high to very high correlations between the volumetric parameters evaluated from 4D flow-cine and bSSFP-cine series. Results for the parameters are given in Table 3 with corresponding scatter plots in Fig. 4 and Bland-Altman plots in Fig. 5. Significant biases were present for all LV volumetric function parameters except the stroke volume; all biases were small (< 5% of the parameter’s mean value) except the ones for LVM. LVM differences between 4D flow-cine and bSSFP-cine evaluation correlated moderately (r = 0.61) with LVM average values, and only LVM (46.2 ± 11.5 g vs. 33.9 ± 8.3 g) showed a significant difference in bias between males and females (*p* < 0.001).

**Table 3:** Relationship of left ventricular and atrial volumetric parameters derived from 4D flow-cine and bSSFP-cine images. r is the Pearson-correlation coefficient between 4D flow-cine and bSSFP-cine parameters. In case of all patients p refers to the significance of the bias of a parameter measured with both methods, in case of the gender comparison p refers to the significance of bias differences between female (*f*) and male (*m*). EDV, end-diastolic volume; ESV, end-systolic volume; SV, stroke volume; LVM, left ventricular mass; EF; ejection fraction; LAV_max_, maximal left atrial volume; LAV_min_, minimal left atrial volume; LATEF, total left atrial ejection fraction

parameter		4D flow-cine	bSSFP-cine	r	bias	*p*
*All*						
EDV (mL)		137.9 ± 28.6	140.8 ± 28.8	0.98	−2.9 ± 5.8	< 0.001
ESV (mL)		46.2 ± 12.7	48.6 ± 13.2	0.96	−2.3 ± 3.8	< 0.001
SV (mL)		91.7 ± 18.4	92.3 ± 18.1	0.95	−0.6 ± 5.8	0.433
LVM (g)		157.8 ± 37.6	118.8 ± 30.6	0.96	39.0 ± 11.4	< 0.001
EF (%)		66.7 ± 4.6	65.8 ± 4.4	0.84	0.9 ± 2.6	0.005
LAV_max_ (mL)		78.6 ± 17.5	77.8 ± 17.9	0.96	0.8 ± 4.4	0.152
LAV_min_ (mL)		36.3 ± 11.1	35.6 ± 12.0	0.97	0.7 ± 3.4	0.143
LATEF (%)		54.2 ± 7.1	54.7 ± 8.1	0.83	−0.5 ± 4.6	0.380
*Gender specific*						
EDV (mL)	*f*	121.4 ± 19.1	125.1 ± 21.0	0.95	−3.7 ± 6.4	0.514
	*m*	161.0 ± 23.1	162.9 ± 23.2	0.98	−1.9 ± 4.8	
ESV (mL)	*f*	39.5 ± 8.9	41.9 ± 9.6	0.93	−2.5 ± 3.6	0.737
	*m*	55.7 ± 11	57.8 ± 12.1	0.94	−2.1 ± 4.0	
SV (mL)	*f*	82.0 ± 13.6	83.2 ± 14.4	0.93	−1.2 ± 5.4	0.334
	*m*	105.3 ± 15.5	105.0 ± 15.0	0.91	0.3 ± 6.4	
LVM (g)	*f*	132.9 ± 20.6	99.0 ± 18.2	0.92	33.9 ± 8.3	0.000
	*m*	192.6 ± 26.6	146.5 ± 21.5	0.91	46.1 ± 11.5	
EF (%)	*f*	67.6 ± 4.9	66.6 ± 4.6	0.85	1.0 ± 2.6	0.653
	*m*	65.5 ± 4.0	64.6 ± 3.9	0.77	0.9 ± 2.6	
LAV_max_ (mL)	*f*	72.9 ± 16.8	72.2 ± 17.6	0.96	0.7 ± 4.7	0.777
	*m*	86.6 ± 15.4	85.6 ± 15.4	0.96	1.0 ± 4.1	
LAV_min_ (mL)	*f*	31.6 ± 8.7	30.8 ± 9.5	0.93	0.8 ± 3.5	0.648
	*mm*	42.8 ± 10.9	42.4 ± 12.0	0.96	0.4 ± 3.3	
LATEF (%)	*f*	56.7 ± 5.2	57.7 ± 5.5	0.66	−0.9 ± 4.4	0.406
	*m*	50.7 ± 7.9	50.6 ± 9.5	0.86	0.1 ± 4.8

### Observer variability

Inter- and intraobserver agreement of LV volumetric function parameters were excellent and did not differ between 4D flow-cine and bSSFP-cine images (Table 4).

**Table 4:** Intraclass correlation coefficients (ICCs) and their 95% confidence intervals in brackets for LV and LA volumetric function parameters evaluated from 4D flow-cine and bSSFP-cine series. EDV, end-diastolic volume; ESV, end-systolic volume; SV, stroke volume; LVM, left ventricular mass; EF; ejection fraction; LAV_max_, maximal left atrial volume; LAV_min_, minimal left atrial volume; LATEF, total left atrial ejection fraction

parameter	Inter-observer variability (*n* = 20)		Intra-observer variability (*n* = 20)	
	4D flow-cine	bSSFP-cine	4D flow-cine	bSSFP-cine
EDV (mL)	0.99	1.00	0.97	0.99
	[0.966–0.994]	[0.988–0.998]	[0.930–0.988]	[0.978–0.998]
ESV (mL)	0.98	0.98	0.97	0.99
	[0.950–0.992]	[0.957–0.993]	[0.926–0.990]	[0.985–0.998]
SV (mL)	0.96	0.99	0.96	0.98
	[0.913–0.986]	[0.969–0.995]	[0.900–0.984]	[0.946–0.994]
LVM (g)	0.99	1.00	1.00	0.99
	[0.984–0.997]	[0.989–0.998]	[0.989–0.998]	[0.977–0.996]
EF (%)	0.87	0.87	0.91	0.89
	[0.708–0.948]	[0.704–0.946]	[0.792–0.963]	[0.738–0.955]
LAV_max_ (mL)	1.00	0.98	1.00	1.00
	[0.985–0.998]	[0.959–0.993]	[0.990–0.999]	[0.989–0.998]
LAV_min_ (mL)	1.00	0.99	1.00	1.00
	[0.988–0.998]	[0.980–0.997]	[0.992–0.999]	[0.988–0.999]
LAVTEF (%)	0.97	0.90	0.97	0.95
	[0.936–0.989]	[0.763–0.959]	[0.936–0.989]	[0.882–0.981]

### Validation of stroke volumes

**Fig. 6:**
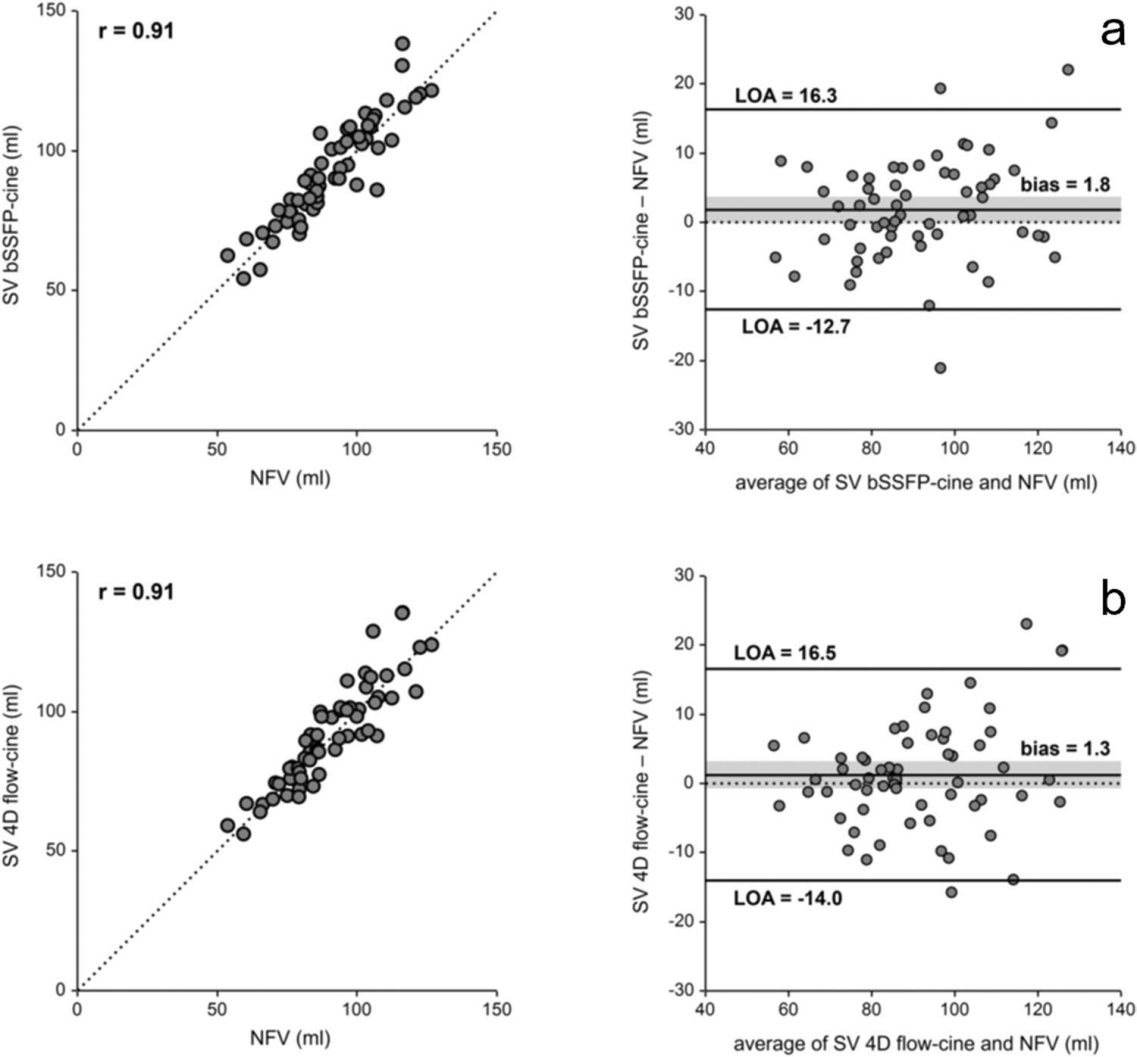
Scatter-plots and Bland-Altman plots comparing pulmonary artery net forward volume (NFV) and stroke volumes determined from 4D flow-cine (**a**) and bSSFP-cine (**b**) images. The dotted line in the scatter plots indicated the line of identity, the grey bar indicates the 95% confidence intervals of bias. r, correlation coefficient; LOA, limits of agreement

Net forward volumes derived from 4D flow imaging correlated very highly with volumetric stroke volumes derived from 4D flow-cine and bSSFP-cine imaging (r = 0.91 in both cases). There was no bias between net forward volume and volumetric stroke volumes (*p* = 0.218 in case of 4D flow-cine imaging, *p* = 0.058 in case of bSSFP-cine imaging). Corresponding Bland Altman plots are shown in Fig. 6.

## Discussion

The main findings of the present study are as follows: 1) LV and LA volumetric function parameters can be evaluated from multiplanar reformatted native multislice 4D flow magnitude images using standard software for cardiac evaluation; 2) all LV and LA volumetric function parameters derived from 4D flow-cine imaging showed high to very high correlations with corresponding bSSFP-cine-derived metrics; 3) LV SV, LA volumes as well as LATEF did not differ between 4D flow-cine imaging and bSSFP-cine imaging; and 4) SVs derived from both of the latter techniques did not differ from NFV derived from 4D flow.

While the assessment of ventricular volumetric function and myocardial mass from contrast-enhanced 4D flow magnitude data has been reported, the assessment of LA volumes and function from native multislice 4D flow magnitude imaging has not yet been analyzed. Image quality of 4D flow-cine images was sufficient to evaluate LV and LA volumetric parameters with excellent inter- and intra-observer variability, comparable to bSSFP-cine images.

Comparison of LV volumetric function parameters derived from multislice 4D flow magnitude and bSSFP-cine imaging showed strong correlations, similar to those found in studies evaluating LV volumetric function parameters from contrast-enhanced 4D flow magnitude data [7–11]. In accordance with those studies, LV SV did not differ between techniques; however, unlike studies employing contrast-enhanced 4D flow, the current study indicates that when using native multislice 4D flow, LV volumes were slightly underestimated and LV EF was slightly overestimated, while LVM was considerably overestimated. It can be speculated that the observed biases are most likely due to FLASH- based 4D flow magnitude data. Overestimation of LVM by up to 19% based on native 2D cine FLASH as compared to bSSFP-cine imaging was previously observed and was attributed to the lower contrast in FLASH images, which was thought to impair differentiation of endocardial voxels containing blood or epicardial voxels containing fat from myocardium [15, 28, 29]. The larger bias in our study might have been caused by the in-plane resolution of the reformatted magnitude cine images being lower than that of the 2D cine FLASH images used in the above-mentioned studies. This aspect might be less relevant for contrast-enhanced data.

In the present study LA volumes as well as LATEF were highly correlated between the two techniques and did not differ significantly. Unlike the LV myocardial wall, the LA wall is smooth and allows clear and reproducible delineation on reformatted 4D flow series at least comparable to that possible on bSSFP-cine series. Biplanar area-length assessment of LA volumetric function especially benefits from retrospective reconstruction, enabling optimized LV 2-chamber and 4-chamber view cut planes [30]. In the present study, angulation of bSSFP-cine long-axis images was thoroughly inspected after acquisition and optimized if necessary—a step that might be overlooked in routine clinical cardiac MR investigations.

As our study cohort was free from cardiac shunts or significant valve regurgitations, mass conservation could be employed for validation of stroke volumes [6, 31]. Both volumetric stroke volumes demonstrated very high correlation to 4D flow-derived NFV with no bias, which can be interpreted as a reciprocal validation between free-breathing bSSFP-cine volumetry and 4D flow velocity data as well as an additional validation of the 4D flow-cine volumetry. Interestingly, there was no difference in the correlation between bSSFP-cine and 4D flow SV with the 4D flow-derived NFV, although the 4D flow results were derived from the same measurement and a poorer correlation between bSSFP-cine SV and 4D flow NFV could be expected due to their sequential measurement. The fact that there was no difference in heart rate between measurements (and also no change in respiratory state) could be interpreted as indication that there was no substantial difference in the physiological state as commonly observed between different sequences due to patient adaption to the scanner environment, discomfort, nervousness or the application of contrast agent between measurements [13].

Some limitations of the present study must be addressed. The investigated population consisted of individuals without symptoms of cardiovascular disease, and image quality and/or applicability of mass conservation could differ for individuals with arrythmia. Similarly, patients without arrhythmia might demonstrate different results, although comparisons between cines with bSSFP- and FLASH readout suggest that the current volumetric results can be transferred to these situations [32]. The velocity encoding of the 4D flow measurement was optimized for intracardiac blood flow, such that the aorta was not free of aliasing in most cases and the pulmonary artery NFV had to be used for mass conservation analysis. However, this has the particular advantage of including coronary blood flow [33] and should therefore yield measurements even better comparable to volumetric stroke volumes. Notability the choice of velocity encoding does not have a direct effect on the 4D flow magnitude images [20] such that the observed volumetric results should not change when choosing a higher velocity encoding. Respiratory gating was not available for the employed multislice 4D flow sequence. Therefore, two-fold averaging was used to compensate for the breathing motion, which might have affected image quality. Image quality was, however, adequate, and moreover, 4D flow without respiratory gating provides higher signal-to-noise ratio on magnitude images and has been shown to be accurate [6]. The free-breathing bSSFP-cine realtime imaging that was used as the standard of reference typically has lower spatial resolution than the standard 2D-segmented bSSFP-cine technique. However, breath-holding might have impacted volumetric function parameters [34], guidelines recommend cine realtime imaging for LV functional assessment in patients who cannot hold their breath [3, 35], and realtime functional parameters have been validated against the 2D segmented approach [36].

In conclusion, native multislice 4D flow magnitude data allows precise evaluation of LV and LA volumetric parameters; however, apart from SV, LV volumetric parameters demonstrate bias and need to be referred to their respective normal values. The evaluation can be performed with standard software and may allow native multislice 4D flow to be applied as a one-stop-shop method of functional cardiac MR imaging, providing consistent, simultaneously acquired volume and flow data.

## Abbreviations

2D: Two-dimensional
3D: Three-dimensional
4D flow: Time-resolved, three-dimensional, three-directional phase contrast imaging
BSA: Body surface area
bSSFP: Balanced steady-state free precession
ECG: Electrographically
ED: End-diastole
EDV: End-diastolic volume
EF: Ejection fraction
ES: End-systole
ESV: End-systolic volume
FLASH: Fast low angle shot
ICC: Intra-class correlation coefficient
LA: Left atrium
LATEF: Total left atrial ejection fraction
LAV_max_: Maximal left atrial volume
LAV_min_: Minimal left atrial volume
LV: Left ventricle
LVM: Left ventricular myocardial mass
NFV: Net forward volume
r: Pearson correlation coefficient
SD: Standard deviation
SV: Stroke volume

## Declarations

### Guarantor

The scientific guarantor of this publication is Ursula Reiter.

### Statistics and biometry

One of the authors (Gert Reiter) has significant statistical expertise. No complex statistical methods were necessary for this paper.

### Study subjects or cohorts overlap

4D flow data of 10 subjects of the study population were included in the definition of an algorithm for automated mitral valve vortex ring extraction from 4D-flow MRI (Kräuter C et al., MRM 84 (2020). https://doi.org/10.1002/mrm.28361). The impact of breath-holding on cine realtime volumetric function parameters was analyzed previously from 56 subjects of this study population (Reiter C et al., Eur J Rad 141 (2021). https://doi.org/10.1016/j.ejrad.2021.109756).

### Methodology

• prospective study

• performed at one institution
